# High flexion knee arthroplasty: the relationship between rotational angles and flexion angle after total knee arthroplasty

**DOI:** 10.1007/s12178-014-9215-1

**Published:** 2014-03-23

**Authors:** Masataka Deie, Tomoyuki Nakasa, Goki Kamei, Atsuo Nakamae, Mitsuo Ochi

**Affiliations:** 1 Institute of Biomechanical and Health Sciences, Laboratory of Musculoskeletal Functional Research and Regeneration, Hiroshima University, 1-2-3 Kasumi, Minami-ku, Hiroshima, Japan 734-8553; 2 Department of Orthopaedic Surgery, Hiroshima University, 1-2-3 Kasumi, Minami-ku, Hiroshima, Japan 734-8551

**Keywords:** Total knee arthroplasty, Rotational angle, Flexion angle, PS type

## Abstract

The current patients required high flexion total knee arthroplasty (TKA). We hypothesized the patients who would get the high rotational angle just after TKA could gain high flexion knee 1 year after TKA. Sixty-three patients (average age: 70.6 years) were examined. In order to examine between the intraoperative rotational angles and the gained flexion angles after TKA, the patients were divided into two groups: the H group (more than 120°) and the L groups (less than 120°) by the gained flexion angles. The relationship between the flexion angles at 1 year after surgery and the intraoperative rotational angle had no significant correlation. But the rotational angles in the L group tended to be higher than the ones in the H group, and at only 30°, the L group gained significantly more rotational angle than the H group. These results might show that a tighter rotational stability induces a gain of higher flexion knee after TKA.

## Introduction

Total knee arthroplasty (TKA) has been widely performed since the 1970s, and the goal of TKA is to achieve a stable and well-aligned tibio-femoral and patella-femoral joint, aiming at long-term clinical patient satisfaction [1, 2]. TKA is understood to reduce knee pain and to recover the daily activities, and is regarded as a cost-effective treatment for end-stage knee osteoarthritis.

Currently, patients require more flexible TKA, especially in Asian people. We believe that the factors to gain higher flexion angles are three points; one is TKA design, second is accurate component placements, and the last is intraoperative ligament balance and joint gaps. With these points in mind, the high-flexion design for TKA was developed. This design had a posterior cruciate retaining, rotating-platform configuration. However, the clinical results, reported had no difference compared with the standard type. Furthermore, there was also no difference in maximum flexion angle between high flexion design and standard type [3]. On the other hand, to gain high flexion angle after TKA, it is necessary to reduce implant malposition. The implant malposition with conventional instrumentation in TKA can be as high as 20 %–40 %, even in major arthroplasty centers, as reported in the literatures [4, 5]. To limit implant malposition, smart tools such as navigation or patient-specific instrumentation have been developed in TKA [6, 7]. The current reports have shown that these tools induced good and accurate implant placement. But the high accuracy of placement does not induce gaining much higher flexion angle after TKA. Recently, many reports have been published about the intraoperative ligament and joints gaps [8]. Whether equalized gaps at extension and flexion are an ideal balance leading to good clinical outcomes or not is still unclear. These developed components and techniques would affect to reduce outliers.

To gain a higher flexion angle, we focused on the relationship between the rotational angle and flexion angle after TKA. To our knowledge, in English literature, the relationship between internal and external rotational angle and flexion angle after TKA is sparse [9]. In this study, we examined the relationship between the rotational angle after TKA and final flexion angle. We hypothesized that patients who would get a high rotational angle just after TKA could gain high flexion angle 1 year after TKA with conventional procedure.

## Material and Methods

From January 2008 to December 2011, we performed more than 150 TKAs in three hospitals. Of these, 63 patients had TKAs performed at Hiroshima University Hospital. They agreed with the contents of this study and were followed-up for more than 1 year. This study was approved by the Epidemiology Research Ethical Review Board at Hiroshima University. All participants received an explanation of the purpose and nature of the study, and their written, informed consent was obtained.

These patients were examined for femoral tibial angle (FTA) before surgery, the length of the gap and balance, and the maximum rotational and flexion angles using the navigation system during surgery; their flexion angle was also evaluated 1 year after surgery.

We used Scorpio NRG Posterior stabilizer (PS) type (Scorpio NRG; Stryker, USA) for all knees; surgery was performed by a single surgeon in our hospital.

Sixty-three patients were included; 9 males and 54 females. Their ages were 66–86 (average 70.6 ± 19.0) years. Range of motion (ROM) before TKA was from –9.2 ± 7.7° to 121.6 ± 20.0°. They were all diagnosed as osteoarthritis of knees with 60 knees having varus deformity and three knees having valgus deformity. The average degree of FTA before TKA was 184.2 ± 9.5 (151°–212°).

In these patients, we evaluated intraoperative gap, balance, and flexion angle, the internal and external rotational angles with 1.5 nm torque. Then we compared the data to the range of motion at 1-year after TKA. To examine between the intraoperative rotational angles and the gained flexion angles after TKA, we divided two groups by the gained flexion angles. One was the H group, in which patients could flex more than 120°. The other was the L group, in which patients could flex less than 120°.

## Surgical procedure and intraoperative evaluations

### Surgical technique of TKA

In this series, surgery on all knees of the patients was performed by the conventional procedure and implanted with Scorpio NRG PS type.

Skin incision was slightly curved at about 15 cm. The joint capsular was opened by medial parapatellar approach. After the osteophytes were removed, based on modified gap control technique; first the tibia was cut using an extramedullary guide, and the femur was cut using an intramedullary guide. Then the balance and gap were checked using the Balancer (Stryker).The posterior site osteophytes of femur were removed intensively, then the medial capsule with medial meniscus attached was also removed to gain high flexion angle. All patients’ patellae were replaced. Before measurement of the rotational angle, the capsule and skin were closed.

### Intraoperative measurement of rotational laxity

**Fig. 1:**
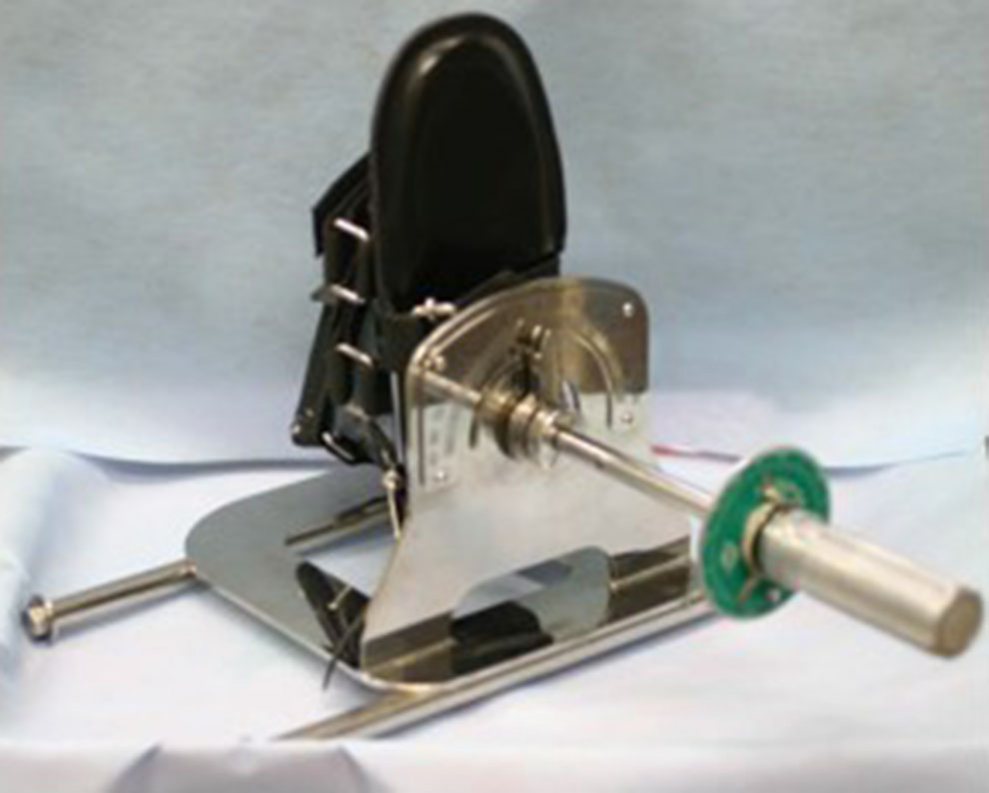
This picture shows our original device

We evaluated the internal and external angles of tibial rotation as the total rotational angles with the knee at 0°, 30°, 60°, 90°, and 120° of flexion after TKA intraoperatively using our original device [10, 11] and navigation system (Orthopilot ACL reconstruction V 2.0; B. Braun Aesculap, Tuttlingen, Germany). This original device has three components, which are the boot, the rotational torque wrench, and the stock (Fig. 1). Fixing the patient’s ankle in this boot prevents rotation of the ankle when a rotational load is applied using the torque wrench. In addition, to keep the femur in the neutral position, we fixed the femur on the operated side to the leg holder, and the assistant surgeon held the femur tightly. This device enabled us to apply a quantitative tibial rotational torque (1.5 nm) equally. The total rotational angles were number of the internal and external angles. We used the navigation system only for the measurement of total range of tibial rotation but not for the placement of femoral and tibial component.

**Fig. 2:**
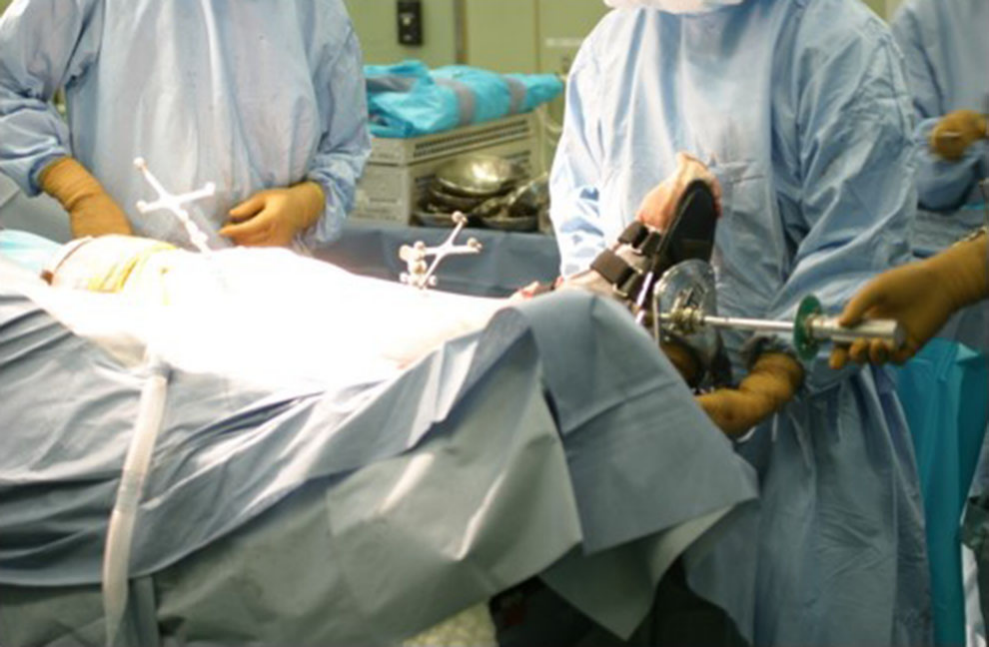
This picture shows the measurement of the rotational angles with our original device and navigation system

To use this navigation system, the femoral and tibial transmitters were fastened to the femur and tibia using special fixation elements with 2 K-wires (2.4 mm each), respectively. Then we registered extra-articular anatomical landmarks, which included tibial tuberosity, the anterior edge of the tibia, and medial and lateral points of the tibial plateau with the pointer. We registered the knee kinematics between 0° and 90°of knee flexion. After these registrations, we fixed the ankle on the operated side to the original device. Then we measured the total range of tibial rotation under the rotational torque of 1.5 nm with the knee at each flexion angle 3 times each after TKA, intraoperatively (Fig. 2).

### Statistical analysis

Pearson’s correlation coefficients were used to explore the relationship between the flexion angle at 1-year after TKA and each parameter, age, preoperative FTA, extension and flexion gap, soft tissue balancing, rotation angle using navigation system, and preoperative ROM. Statistical differences between the H and the L groups were calculated using the Mann-Whitney U test. A *P*-value of less than 0.05 was considered significant with the numbers available.

## Results

All patients had reduced pain and could walk by themselves 1 year after surgery. In these patients, there were no remarkable complications such as infection and clunk syndrome.

### Intraoperative evaluation

The gap at 0° and 90° were 22.8 ± 3.1 mm and 23.3 ± 3.2 mm, respectively. The differences of the absolute value of the gap between 0° and 90° in all cases were within 4 mm and average 1.5 ± 0.4 mm. The balance at 0° and 90° were varus 2.3° ± 3.3° and valgus 0.3° ± 3.4°, respectively. The differences of the balance between 0° and 90° in all cases were within 4°, and average was 1.5° ± 1.1°.

Just after TKA, the rotational angles were measured at 0°, 30°, 60°, 90°, and 120°. The total rotational angles at 0°, 30°, 60°, 90°, and 120° were 4.7° ± 2.4°, 11.5° ± 4.3°, 12.8° ± 4.1°, 8.7° ± 4.8°, and 4.3° ± 4.6°, respectively.

ROM just after TKA was 0.1° ± 1.0° to 128.2° ± 8.7° and ROM 1 year after TKA was –3.0° ± 6.1° to 114.6° ± 12.5°.

The relationships between the ROM at 1 year after surgery and the intraoperative rotational angle at each measured angle had no significant correlation.

With regard to the H group and the L group, the H group consisted of 28 cases and the L group included 35 cases. In the H group, the age was 66–86 years (average 70 years) and their FTA before TKA was 151°–212° (average 184.8°). ROM before TKA was –7° ± 6° to 126.7° ± 14.2°. In the L group, the age was 67–86 years (average 71.2 years) and their FTA before TKA was 172°–201° (average 183.8°). ROM before TKA was –10° ± 8° to 118.7° ± 22.3°. There were no significant differences in operated age, preoperative FTA, and ROM before surgery between the H and the L groups.

**Fig. 3:**
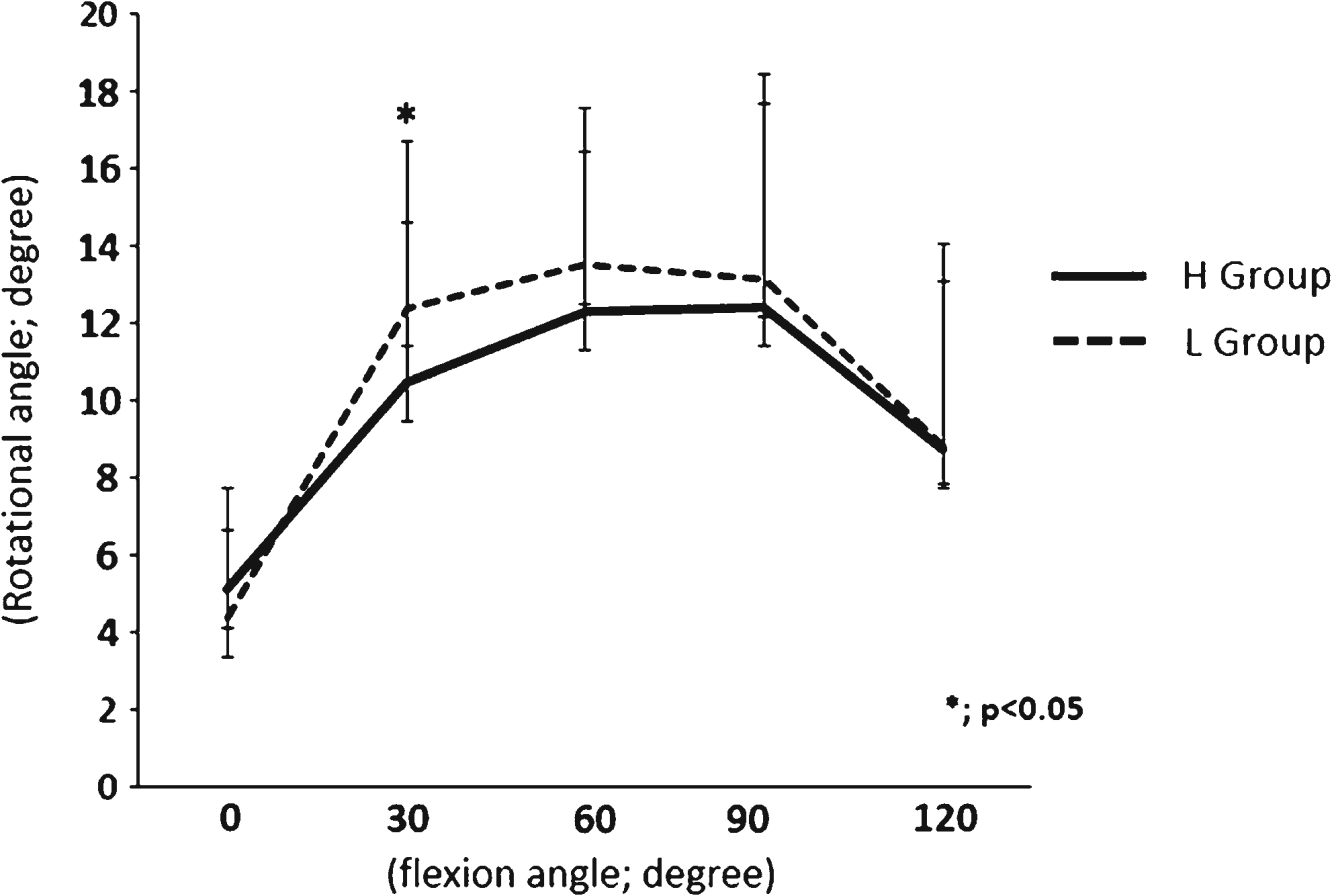
This graph shows the rotational angles of the H and L groups during knee flexion. The rotational angle of the L group was higher than the one of the H group. And at 30° the rotational angle for the L group was significantly higher than the ones gained for the H group

For rotational angles, significant difference at 30° was found between the H and L groups (*P* < 0.05) (Fig. 3). At 30°, the L group gained more rotational angles than the H group. At other angles 0°, 60°, 90°, 120°, there were no significant differences between both groups, whereas rotational angles in the L group were higher than the ones in the H group (Fig. 3).

## Discussion and conclusions

This study examined the relationship between the flexion angle after TKA and the rotational angles just after TKA. Although there are many reports on ROM after TKA, reports of the relationship on rotational angle and gained ROM are sparse.

Knee flexion over 120° commonly has been referred to as deep flexion. Kanekasu et al [9] reported that tibial rotation increased with greater knee flexion, and also described that the greatest internal rotation observed was 17° at 137° flexion using fluoroscopy study with TKA knee. In general, TKA plans to allow 20°–25° for internal and external rotation, respectively, and from the product company data (Stryker) and reports of the Scorpio, NRS PS type allowed internal and external rotation of 25°. However, the total rotational angles from our results did not gain over 15°, even after we applied 1.5 nm under anesthesia. The human nature knee allows about 20°–30° for internal and external rotation, respectively, during knee motion [12], and it is known that the tibial rotation can be 20°–30° for internal and external rotation at 20° or 30° knee flexion. Furthermore, at 90° knee flexion, the tibia can rotate more than 40° for external rotation; Nakagawa et al [13] reported degree of tibial internal rotation from 90° to 162° flexion in MRI analysis of deep flexion in 20 healthy knees. We could describe that TKA knee did not mimic normal knee on rotational angles. However, the changes of available rotational angle from 0° to 120° after TKA, the rotational angles at 60° or 90° knee flexion were maximum and the rotational angles at 0° knee extension were minimum. These results were similar to the rotational angle variation of the healthy knee joint. In this regard, we could say that the TKA knee mimicked the original knee joint on the change of the rotational angle according the knee flexion.

We used to believe that more rotational angle after TKA would bring higher knee flexion 1 year after TKA. But from the current results, our hypothesis was rejected. The patients who obtained fewer rotational angles in the immediate postoperative period showed a larger flexion angle at 1-year after TKA. Especially at 30° knee flexion we found significant differences of the rotational angles between the H and L groups. As for the factors that influence the flexion angle after TKA, medial pivot kinematics during knee flexion is one of the important ones. Nishio et al demonstrated that knee flexion angle in medial pivot pattern TKA was significantly better than that in nonmedial pivot pattern TKA [14•]. Matsuzaki et al showed that postoperative internal tibial rotation was a correlation factor with postoperative knee flexion, and preoperative internal tibial rotation did not change postoperatively [15•]. These reports suggested that appropriate rotational axis during flexion is crucial to obtain high knee flexion after TKA. In our study, intraoperative large rotational angle in the L group may be reflected nonappropriated rotational axis during knee flexion, such as nonmedial pivot kinematics.

This study has some limitations. First, we evaluated total rotational angle instead of internal and external rotational angle. In a normal knee, external rotational angle is larger than the internal rotational angle. However, it is difficult to find a neutral position of tibio-femoral alignment for TKA indicated in patients in intraoperative measurements. Therefore, we used total rotational angle. Second, we evaluated only 1 measurement for rotational angles intraoperatively. We used navigation system for this study. This system requires that a K-wire needs to be inserted and, thus, we could not measure the rotation in an outpatient clinic. In the next step, we will use a measurement tool such as a rotameter [16]. Third, the number of patients was relatively small and the follow-up time was only 1 year.

In conclusion, there was no significant relation between rotational angle and flexion angle. However, the higher flexion knees after TKA had less rotational angles just after TKA. These results might show that a tighter rotational stability was induced to gain a higher flexion knee after TKA.
